# Thermo-Mechanical Characterization of Metal–Polymer Friction Stir Composite Joints—A Full Factorial Design of Experiments

**DOI:** 10.3390/polym16050602

**Published:** 2024-02-22

**Authors:** Arménio N. Correia, Beatriz M. Gaspar, Gonçalo Cipriano, Daniel F. O. Braga, Ricardo Baptista, Virgínia Infante

**Affiliations:** 1 Instituto Superior Técnico, Universidade de Lisboa, 1049-001 Lisboa, Portugal; beatriz.m.g.ribeiro@tecnico.ulisboa.pt; 2 Institute of Science and Innovation in Mechanical and Industrial Engineering, 4200-465 Porto, Portugal; gcipriano@inegi.up.pt (G.C.); dbraga@inegi.up.pt (D.F.O.B.); 3 Instituto Superior de Engenharia de Lisboa, Instituto Politécnico de Lisboa, 1959-007 Lisboa, Portugal; ricardo.baptista@isel.pt; 4 LAETA, IDMEC, Instituto Superior Técnico, Universidade de Lisboa, 1049-001 Lisboa, Portugal; virginia.infante@tecnico.ulisboa.pt

**Keywords:** friction stir joining, composite joints, joint morphology, mechanical strength, processing temperature, full factorial design

## Abstract

With the increasing demand for lighter, more environmentally friendly, and affordable solutions in the mobility sector, designers and engineers are actively promoting the use of innovative integral dissimilar structures. In this field, friction stir-based technologies offer unique advantages compared with conventional joining technologies, such as mechanical fastening and adhesive bonding, which recently demonstrated promising results. In this study, an aluminum alloy and a glass fiber-reinforced polymer were friction stir joined in an overlap configuration. To assess the main effects, interactions, and influence of processing parameters on the mechanical strength and processing temperature of the fabricated joints, a full factorial design study with three factors and two levels was carried out. The design of experiments resulted in statistical models with excellent fit to the experimental data, enabling a thorough understanding of the influence of rotational speed, travel speed, and tool tilt angle on dissimilar metal-to-polymer friction stir composite joints. The mechanical strength of the composite joints ranged from 1708.1 ± 45.5 N to 3414.2 ± 317.1, while the processing temperature was between 203.6 ± 10.7 °C and 251.5 ± 9.7.

## 1. Introduction

According to the European Environment Agency, the current transport model exhibits a major downside by being responsible for about a quarter of the EU’s total greenhouse gas emissions that accelerate the climate change crisis. For this reason, in seeking a climate-neutral EU by 2050, the decarbonization of the mobility sector has become a priority within political and industrial agendas, demanding the adoption of alternative propulsion systems to replace fossil fuel-based methods [1].

To this end, battery-based electric vehicles (BEVs) and hydrogen fuel cell vehicles (hFCVs) have become prominent alternatives, with extensive research and investment in recent years [2,3,4]. Nevertheless, given the early stage of technological development, significant challenges need to be addressed to fully embrace such propulsion systems. On the one hand, and despite recent improvements, BEVs still exhibit low energy density in their batteries when compared with fossil fuels, leading to heavier vehicles than internal combustion engine (ICE) ones and jeopardizing the autonomy/range as well as the dynamic performance [5,6,7]. Additionally, the thermal management of battery systems is another key factor that directly influences BEV performance since operating temperatures out of the optimal range lead to a significant decrease in range/autonomy [8,9]. On the other hand, in hFCVs, H_2_ storage requires extreme conditions such as very high pressure or very low temperatures, respectively, depending on whether it is stored in a gas or liquid state [3,10]. Furthermore, metallic reservoirs are also prone to H_2_ embrittlement with significant deterioration of both their static and fatigue properties [11,12]. These technical requirements result in a significant weight increase in H_2_ metallic containers in order to ensure a safe and durable operation [13].

To overcome these challenges, new integral multi-purpose designs are required; therefore, dissimilar structures offer the ability to take advantage of each material’s strengths, becoming desirable in today’s reality. Even though dissimilar structures are advantageous and desirable, the inherent differences in physical, mechanical, and chemical properties of the base materials cause new challenges during joining processes, and the development of suitable manufacturing technologies is now, therefore, required [14].

One line of research that might tackle these difficulties is solid-state joining, in which the drawbacks associated with melting and re-solidification processes are mitigated, resulting in joints with superior properties [15,16,17]. In the 1990s, friction stir welding (FSW) technology was applied to the manufacturing of fuel tanks and later fitted on satellite launch vehicles, exhibiting remarkable results in terms of improved joint strength, with reduced costs associated with manufacturing operations [18].

FSW is a solid-state welding process, and, for that reason, the issues commonly associated with re-solidification in fusion welding processes such as cracking, excessive softening of the heat affected zone (HAZ), distortion, and/or residual stresses, can be minimized. During the welding process, the mechanical energy supplied by the tool is converted into heat through friction between the surface of the tool and the base materials. Moreover, the energy released by the materials undergoing severe plastic strain is also converted into heat, and both mechanisms generate the necessary heat input to produce the desired joint; therefore, it is considered a clean and environmentally friendly technological process [15]. The FSW process is commonly divided into the following four stages: (i) the plunging stage, (ii) the dwelling stage, (iii) the welding stage, and (iv) the retracting stage [19]. The industrial application of such technology is not confined to the aeronautical/space industry, with the automotive industry also showing interest in applying FSW technology to the manufacturing processes associated with battery trays [20,21], battery packs [22], and the fuel cell system’s subframe [23].

Enabling the design and manufacturing of integral dissimilar structures extends the scope of applicability and also its impact. A considerable amount of research on FSW and its industrial applications has been associated with aluminum alloys and, in recent years, the use of FSW to join metal alloys and polymer matrix composites has also taken a step forward. Following industrial trends and needs, a wide array of successful research has been carried out on joining different sets of metal alloys and polymers/composites. The reported research has shown that the main binding mechanisms developed between such dissimilar materials using friction stir-based technologies are mechanical interlocking and chemical adhesion, in which the tooling geometry and heat input control were shown to be key factors in the enhancement of the joints’ mechanical behavior [24,25,26,27,28,29,30,31,32].

Derazkola et al. [33] successfully joined a 5000 series aluminum alloy and polymethyl methacrylate in an overlap configuration. The morphological features were identified as the primary binding mechanism, enabling mechanical interlocking between base materials. Moreover, chemical bonding along the joints’ interface promoted a secondary bonding mechanism, enhancing the joints’ strength. Using the same configuration, Ratanathavorn and Melander [34] used friction stir welding to join a 6000 series aluminum alloy and polyphenylene sulfide. The joining region showed a blend of aluminum chips inserted within the polymeric matrix, and the mechanical interlocking was pointed out by the authors as the primary joining mechanism.

In order to explore the feasibility of joining a magnesium alloy and polyethylene, Liu et al. [35] used friction lap welding (FLW) as a joining technology. Pre-joining treatments such as corona discharge and plasma electrolytic oxidation were used on the surfaces of the polymeric and metallic sheets. The main binding mechanism involved chemical bonding between base materials through the induced oxidation interlayer created by the pre-treatment. The polymer was kept embedded within the oxidation interlayer, creating mechanical interlocking at the micro-scale. In the same way, Okada et al. [36] investigated composite joining between metals and polymers using FLW. In their research, they attempted to join a 2000 series aluminum alloy with ethylene–acrylic acid copolymer (EAA) and high-density polyethylene (PE). When using the metallic sheet in the as-received condition, the authors were only able to join it with EAA, although after anodizing the metallic sheet, it was possible to join it with both polymers. The main difference was in the presence of a functional group in the EAA polymeric chain, in contrast with PE. This discrepancy was indicated as the main reason behind the different joinability between material pairs when using the aluminum alloy in the as-received condition. After anodizing, the interface revealed polymeric material physically attached to the oxide film, inducing the mechanical interlocking effect at the micro-scale, and this was in line with the observations and conclusions of Liu et al. [35].

Friction stir-based technologies enable the fabrication of composite joints with mechanical strengths that may vary significantly, given that the used base materials also exhibit substantially different mechanical properties. Consequently, the comparison among published studies is usually accomplished by using the joint efficiency, the ultimate tensile strength (UTS) ratio between the joint and the weakest base material, as a benchmark. Table 1 presents a summary of the results obtained in this field.

The fatigue behavior of dissimilar friction stir joints is heavily dependent on the existence of defects such as voids and hooks, particularly in the case of overlap joints, since these defects act as stress raisers, inducing crack nucleation over time. Furthermore, it was reported that the presence of micro-defects also increases fatigue crack growth in dissimilar joints due to the development of secondary cracks [14]. Although joining dissimilar materials remains a challenge, friction stir-based technologies show potential to overcome it through process improvements toward enhancing fatigue and impact strength in addition to static strength, which are prerequisites for the widespread adoption in BEVs and hFCVs.

In this research study, the full factorial design method and ANOVA were used to explore the impact of the tool tilt angle and the rotational and travel speeds on the mechanical strength and processing temperature of composite joints manufactured with a friction stir joining (FSJ)-based technology. The composite interface was subjected to a comprehensive analysis by assessing the hardness profile as well as the macro- and microstructures of the cross-section of the fabricated joints.

## 2. Materials and Methods

An engineering-grade polymer, Noryl^TM^ GFN2, and an aluminum, magnesium, and silicon alloy, AA6082-T6, were joined using a friction stir-based technology. Noryl^TM^ is a family of polymers based on modified polyphenylene ether resin with high-impact polystyrene. Depending on the grade, it may be reinforced with glass fibers to further improve the inherent physical and chemical attributes, as is the case of the GFN2 grade, which incorporates 20 wt. % of short glass fibers. The key attributes of this material are associated with its high heat resistance and dielectric strength, excellent dimensional stability, good processibility, and low density, offering a good balance of mechanical and chemical properties. AA6082-T6 is a medium-strength aluminum alloy that offers good machinability and weldability while exhibiting superior corrosion resistance. The main physical and thermo-mechanical properties of the base materials can be found in Table 2.

To assess the main effects and interactions of tilt angle and rotating and travel speeds on the mechanical strength and processing temperature of friction stir composite joints among such base materials, a full factorial design of experiments was outlined, as summarized in Table 3.

**Figure 1:**
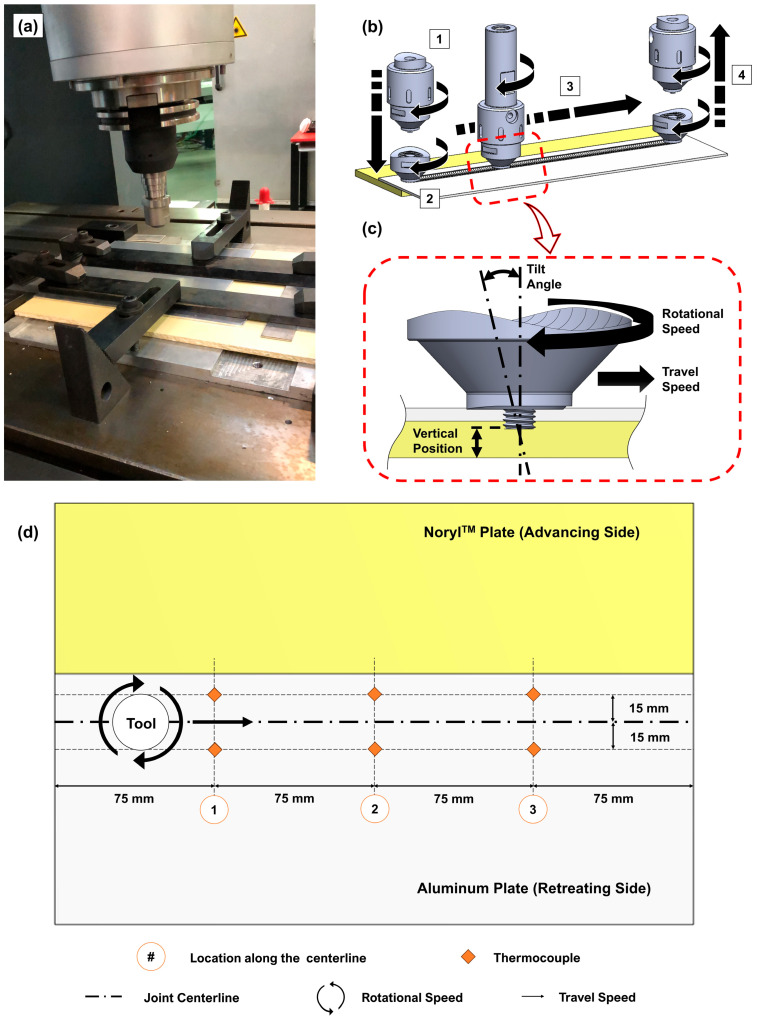
(**a**) Experimental procedure setup and schematic of (**b**) the schematic of process: 1—plunging stage, 2—dwelling stage, 3—joining stage, and 4—retracting stage, (**c**) side view detail with the main processing parameters, and (**d**) top view with the thermocouples’ relative position (not to scale).

The aluminum and polymeric plates, with 2 and 5 mm of nominal thickness, respectively, were cut into 300 × 125 mm plates and positioned with an overlap of 40 mm. The composite joints were fabricated using a custom-built FSW machine equipped with a modular tool composed of a 2 mm long and 5 mm diameter threaded cylindrical probe attached to a flat shoulder with outdented screw features and a 16 mm diameter. Both components were manufactured using AISI1045, a medium-carbon steel, so that proper load transfer and geometrical stability were ensured throughout the joint fabrication process. The tool path was set to be centered within the overlapping region. The polymeric plate was positioned on the advancing side (AS) of the joining centerline underneath the aluminum plate, as depicted in Figure 1. The fabrication process was position-controlled with a constant vertical position of 4.9 mm above the backing bar (refer to Figure 1 for further details).

Considering the torque and forces induced on the base materials, the clamping system was also designed to ensure their relative position while providing a stable setup during the joining procedure. To measure the thermal history throughout the joining processes, 6 k-type thermocouples were installed on the upper surface of the aluminum plates and connected to a National Instruments^TM^ c-series 9181 Ethernet chassis (Austin, TX, USA) with an acquisition rate of 70 Hz. To record the thermal measurements, NI SignalExpress 2013 software was used. The thermocouples were positioned 15 mm to each side of the joining path and were 75 mm away from each other, as schematized in Figure 1.

After the joints were fabricated, they were cut perpendicularly to the joining centerline into five specimens, three of them were 25 mm wide, and the remaining two were 5 mm wide. The 25 mm wide specimens were subjected to quasi-static tensile shear testing using the Instron^TM^ 5566 universal testing machine (Norwood, MA, USA). The 5 mm specimens were cold mounted, polished, and etched, with each one of the samples being subjected to scanning electron microscopy (SEM) with energy dispersive spectroscopy (EDS) and microhardness testing. The SEM/EDS analyses were performed using an analytical SEM Hitachi^TM^ S2400 (Tokyo, Japan) with a Bruker light elements EDS detector (Billerica, MA, USA), whereas the microhardness tests were performed on a Shimadzu^TM^ HMV-2 machine (Kyoto, Japan).

## 3. Results and Discussion

A full factorial design of experiments is a methodical approach that systematically analyzes the effects of multiple factors and their interactions on response variables. This methodology enables a comprehensive analysis of all possible combinations of factors levels, ensuring that no significant main effects or interactions are overlooked within the region under investigation. The use of a FFD of experiments leads to accurate and reliable conclusions about the factors’ impact on the response since the experimental data are subjected to an analysis of variance, which provides quantitative metrics over the significance of each factor and their interactions [39].

A two-level FFD (2^k^) with three factors (k = 3) was designed to assess the influence of joining parameters on the mechanical strength and processing temperature of aluminum–polymer friction stir composite joints. Since the DoE was designed with three factors (tilt angle, rotational speed, and travel speed) and two levels, a total of eight (2^3^ = 8) composite joints with different parameter settings were fabricated. The range associated with each independent variable under analysis was based on previous research carried out by Correia et al. [19,40]. The numerical expressions that relate the joining parameters to both mechanical strength and processing temperature are expected in the form of Equation (1).

(1) $$Y=\beta_{0}+\beta_{1}X_{1}+\beta_{2}X_{2}+\beta_{3}X_{3}+\beta_{12}X_{1}X_{2}+\beta_{13}X_{1}X_{3}+\beta_{23}X_{2}X_{3}+\beta_{123}X_{1}X_{2}X_{3}+\epsilon$$

Since an FFD with two levels will always provide an apparent linear relationship between the independent and dependent variables, and to experimentally confirm the fitness of the obtained FFD mathematical model, an additional composite joint (J9) was fabricated using the central values of the domain volume under analysis.

### 3.1. Mechanical Strength

Table 4 lists the processing parameters and levels used to fabricate each composite joint, as well as the average ultimate tensile load (UTL) obtained for each configuration. The lowest mechanical strength was obtained in joint J7 with an average UTL of 1708.1 ± 45.5 N, which was approximately half of the mechanical strength exhibited by joint J4, with an average UTL of 3414.2 ± 317.1 N. From the tensile shear strength test results, it was also noticeable that an increase in joint strength also implied an increase in the standard deviation of the results, with joint J4 exhibiting the most dispersed results, whilst joint J7 evidenced the second lowest standard deviation of mechanical strength.

To obtain a mathematical relationship between the processing parameters and mechanical strength, the results were subjected to an analysis of variance (ANOVA). The analysis was conducted with a confidence interval of 95% to determine the significance of the statistical model and its terms. The significance of each term was determined by assessing the F- and *p*-values, which are listed in Table 5.

The terms with a *p*-value over 0.05 indicate that they are not significant, and for that reason, the independent parameter travel speed and the two-way interaction between the tilt angle and rotational speed were found to be non-significant. As expected, the remaining independent variables and interactions were significant, with the three-way interaction term exhibiting the largest contribution (25.7%) to the mechanical strength of the composite joints. The tool tilt angle and its two-way interaction with the travel speed contribute 20 and 25.4%, respectively. Thus, these three terms account for over 70% of the UTL of the fabricated joints. In what refers to the fitness of the model, the determination coefficient (R^2^) was found to be 0.908, meaning that around 90% of the variance in the UTL can be explained by the independent variables (ω, v, and α). Additionally, the adjusted value (AdjR^2^ = 87.5%) indicates a good fitness of the model, being close to 90%.

**Figure 2:**
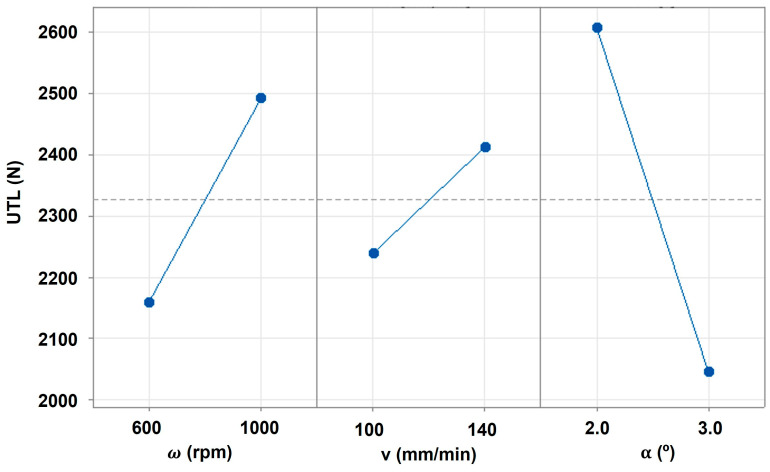
Main effects of the joining parameters on UTL.

**Figure 3:**
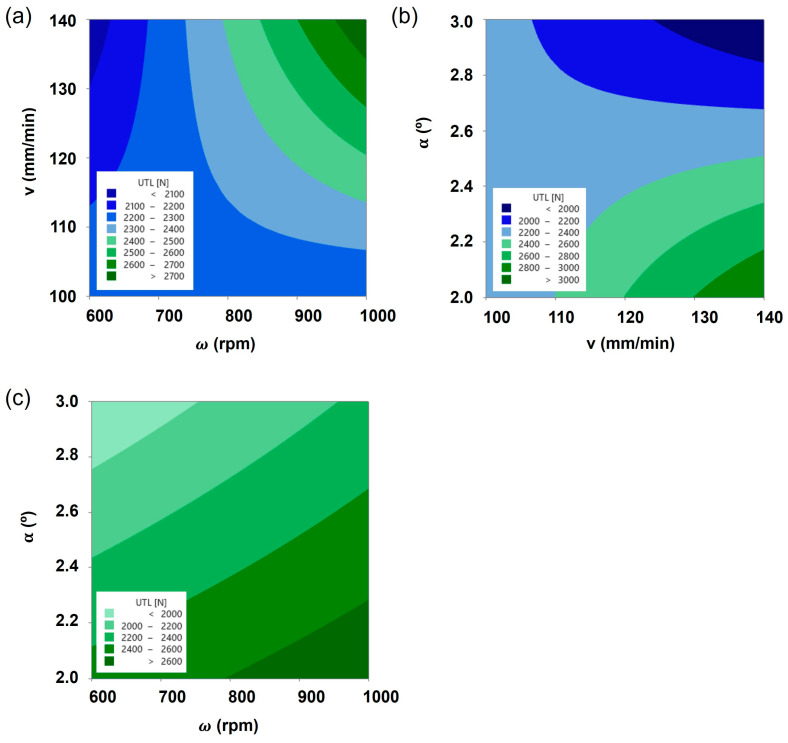
Contour plots of UTL as a function of interactions between (**a**) rotational speed and travel speed, keeping a 2.5° tilt angle, (**b**) travel speed and tilt angle, keeping 800 rpm of rotational speed, and (**c**) tilt angle and rotational speed, keeping 120 mm/min of travel speed.

The main effect plots on UTL are displayed in Figure 2, and it is observable that, contrarily to the rotational and travel speeds, the tool tilt angle exhibits a negative relationship with the composite joint strength, denoted by the negative slope of the plot effect. This means that within the range of analysis, a decrease in tool tilt angle is expected to increase the joint strength, whilst increasing rotational and travel speeds induce a positive effect on joint strength, with each one of the parameters having its own contribution, as discussed previously. By analyzing the interaction contour plots displayed in Figure 3, the non-linear behavior of the composite joint strength is observable, confirming a significant interaction among the processing parameters. On the one hand, the interaction between travel and rotational speed revealed a wide saddle-like response for lower values of those parameters. In this region, the composite joint strength is lower yet less sensitive to changes in those parameters, explaining the significant reduction in the variance in the response identified in Table 4. When analyzing the interaction between tilt angle and travel speed, it is also observable that a wide saddle-shaped response for low values of travel speed leads to lower values of UTL. As identified previously, the variance in this region is low, and the response is less sensitive to small changes in the joining parameters. On the other hand, higher levels of the UTL response are found in the corners of the regions under analysis, and, due to this fact, they will exhibit a significant amount of variance considering the increased sensitivity to small changes in the operating conditions.

Additionally, given the shape of the main effects and interactions plots, it seems that the optimum level would be close to the values obtained with joint J4’s parameters. Yet, the optimum region is very narrow (as evidenced in Figure 3), which may lead to increased volatility in the results, as occurred in the results obtained with joint J4.

Taking into consideration all the above, the mathematical model that relates the composite joints’ UTL and the processing parameters, with a confidence interval of 95%, is given by Equation (2).

(2) UTL=36744−53.78·ω−275.4·v−12286·α+0.4485·ω·v+19.4·ω·α+95.6·v·α−0.159·ω·v·α

Using the mathematical model expressed in Equation (2), the predicted UTL for the joints fabricated with the parameters associated with the validation joint (J9) is 2333.0 N. Since the average UTL of joint J9 was found to be 2350.0 ± 192.4 N, the error between the predicted value and the experimental one was 0.73%. This way, the accuracy and predictability of the model could be considered experimentally validated.

### 3.2. Processing Temperature

The processing temperature during the FSJ process is one of the key factors enabling the fabrication of sound metal–polymer composite joints. The temperature build-up predominantly originates from two thermo-mechanical effects: (a) heat generation due to friction between the tool (shoulder and pin) and the base materials and (b) heat release due to base materials’ plastic flow [40,41,42].

The processing temperatures were measured both on the AS and RS of the joints, and the average values are listed in Table 6. As observed by Correia et al. [40] and Silva et al. [43], the average processing temperature is higher on the advancing side, which is a behavior that can be explained by a more intense stirring flow on this side of the processing centerline.

Taking into consideration that the fabrication of friction stir composite joints implies important levels of thermo-mechanical processing of the base materials, the assessment of the process temperature within the joining regions becomes extremely difficult. For that reason, the average temperature at the advancing side was considered a proxy of the processing temperature. The mathematical relationship between the joining parameters and the average processing temperature on the AS was determined by carrying out an ANOVA with a 95% confidence interval. The significance of each term and its contribution to the average advancing side temperature is summarized in Table 7.

For enhanced accuracy of the model, a backward elimination of the terms with a cut-off value of 0.1 was incorporated, and consequently, the terms with *p*-values larger than this value were disregarded. From the resulting statistical model, the independent variables, i.e., tilt angle and rotational speed, were found to be the ones with the largest contribution to the average AS temperature, with 55.0 and 24.7%, respectively, accounting for almost 80% of the contribution.

In terms of the fitness of the model, the R^2^ was found to be 93.7%, while the adjusted R^2^ was found to be 87.3%, both indicating the good fitness of the model. Henceforth, the numerical expression that relates the average temperature on the AS and the processing parameters can be found in Equation (3).

(3) Tas=381.1+0.03896·ω−0.991·v−79.4·α+0.467·v·α

**Figure 4:**
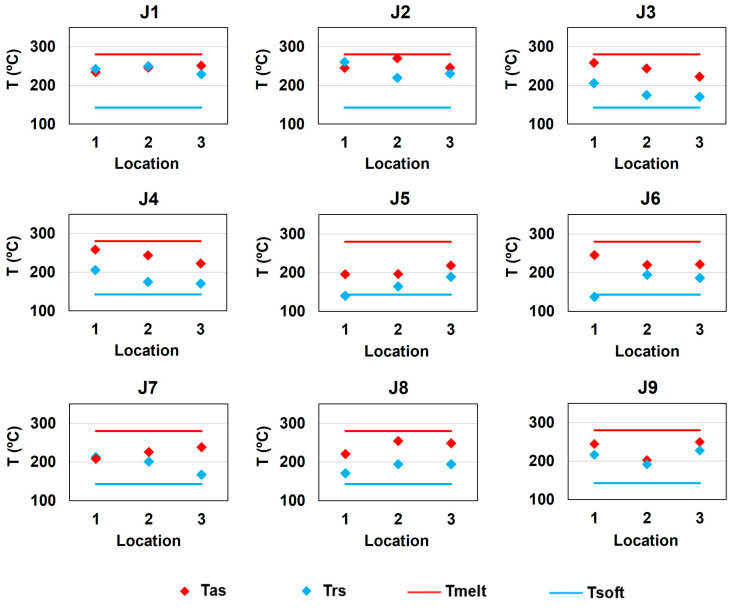
Composite joint processing temperature on the advancing side (Tas) and retreating side (Trs).

In this way, and in accordance with the statistical model, the predicted average temperature on the AS during the joining process with the parameters of joint J9 is 237.6 °C. Compared with the average experimental value of 232.2 °C displayed in Table 7, the error is found to be 2.3%, which experimentally validates the statistical model. When further analyzing the thermal history of the fabricated composite joints, as displayed in Figure 4, it was also distinguishable that despite the significantly different performance evidenced by each joint, the recorded temperatures were found to be between the softening and melting temperatures of Noryl^TM^.

This evidence corroborates the hypothesis proposed by Correia et al. [40], who state that the polymer softening temperature acts as the minimum processing temperature required to enable the proper development of binding mechanisms, while processing temperatures substantially higher than the melting temperature may disable the joining ability of the process due to polymeric overflow. On the one hand, all the tested specimens failed at the polymeric end of the joints rather than through the joints’ interface, and, on the other hand, none of the specimens showed polymeric overflow. These themes will be further analyzed subsequentially in this paper.

### 3.3. Hardness

**Figure 5:**
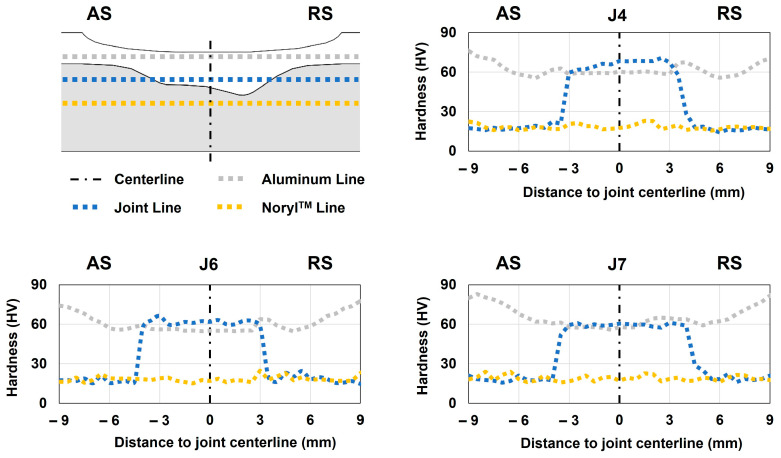
Microhardness testing scheme and results for joints J4, J6, and J7.

The analysis of the microhardness profile was performed in joints J4, J6, and J7 since they exhibited the maximum, intermediate, and lowest mechanical strengths, respectively. Given that we are analyzing a metal–polymer composite joint, the measurements were taken in three different regions: (i) the aluminum line, (ii) the joint line, and (iii) the Noryl^TM^ line, as schematized in Figure 5. The hardness of the base materials was previously assessed to define the baseline values for the analysis. This way, the hardness values were found to be 102 HV_1_ and 18 HV_0.2_ when testing the aluminum and Noryl^TM^ plates, respectively.

After examining the hardness plots displayed in Figure 5, it could be concluded that the change in processing parameters did not have a relevant effect on this attribute. The hardness profile measured over the aluminum line (which only covers the aluminum side of the joint) evidenced a W shape that was also observed by several authors [44,45,46]. This shape is a characteristic associated with heat-treatable aluminum alloys, such as the present case, when subjected to friction stir processes [44]. These profiles depict a noticeable drop in hardness within the thermo-mechanically affected zone (TMAZ) and stirring zone (SZ) of the aluminum portion of the joint, even with small differences in the recorded hardness values in the different joints. Compared with the values measured on the aluminum portion over the joint line (the line that covers both the aluminum and polymer), the hardness was found to be slightly higher and in line with the W-shaped profile characteristics. Regarding joint J7, the hardness values were found to be almost the same when comparing the aluminum line and joint line. One reason that might explain this behavior is a more homogeneous grain size within the stirring region in joint J7, whereas joints J4 and J6 might have a finer grain toward the joint line, which will lead to increased hardness within this region. Regarding the measurements taken over the polymeric portion of the joint, the values were found to be stable around the nominal value, regardless of the region and joint under analysis.

The three obtained profiles revealed a substantial decrease in hardness at the aluminum portion of the joints, indicating that the metallic alloy lost the hardening properties associated with the T6 heat treatment of quenching. As a result, it is expected that the strength of the aluminum plate in these regions also decreased, as suggested by Costa et al. [44]. Nevertheless, this foreseeable decrease in the mechanical strength of the aluminum did not have a relevant impact on the joints’ strengths for two main reasons: (i) the major difference in mechanical strength between both base materials (even without the quenching hardening properties) and (ii) the failure of the joints took place at the polymeric end of the joints in all the tests, as analyzed in the following section.

### 3.4. Macro- and Microstructure

The visual inspection of the fabricated composite joints revealed a superior surface finish with well-defined onion rings and exit holes, which are key geometrical features associated with friction stir-based technologies [15,47]. Nonetheless, some neglectable flash was observed in joints J1 and J7.

**Figure 6:**
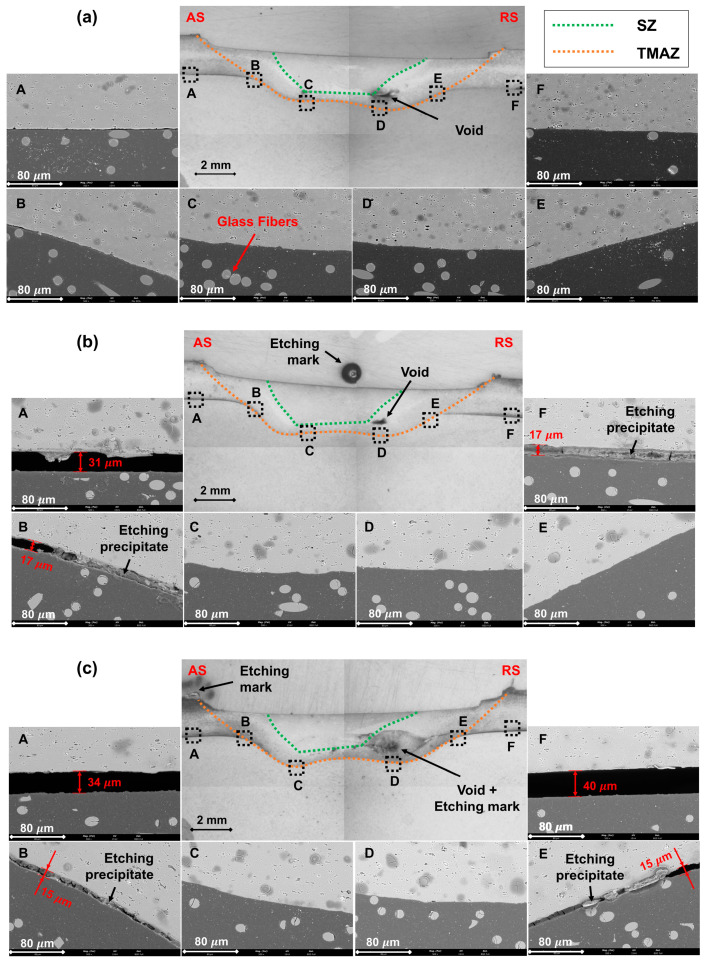
Cross-sectional macro- and microstructures of (**a**) Joint J4, (**b**) Joint J6, and (**c**) Joint J7.

With the purpose of identifying morphological characteristics that might explain the differences in mechanical performance among the composite joints, a thorough analysis of the macro- and microstructures of joints’ cross-sections was accomplished. To comprehensively characterize the composite joints’ interfaces, SEM/EDS analysis was performed in six distinct locations on the previously selected joints—J4, J6, and J7—as schematized in Figure 6.

The macrostructure of the joining region of the three joints was found to be remarkably similar, with the aluminum plate being clinched into the polymeric plate. The interface between the base materials evidenced a prominent double concavity shape, which is a geometrical feature that induces macro-mechanical interlocking between the base materials, as previously reported by Correia et al. [19,40]. All three joints exhibited a small void located between the stirring zone (SZ) and the thermo-mechanically affected zone (TMAZ) toward the RS of the joints; this defect was also identified and explained by Derazkola et al. [27,33,38,48] and Huang et al. [28,49]. Even so, these defects did not directly affect the mechanical performance of the joints, as all of them failed outside the joining region.

Contrarily, the microstructural examination revealed significant differences among the joints under analysis. On the one hand, the joint with the highest mechanical strength, J4, evidenced a smooth transition between the base materials in all six locations along the interface perimeter (see Figure 6 for further insights), meaning that the effective and apparent joining areas were the same. On the other hand, the joint with intermediate mechanical strength, J6, revealed micro-gaps in positions A, B, and F, while the joint with the least mechanical strength, J7, revealed similar gaps in positions A, B, E, and F, as depicted in Figure 6. For this reason, joint J7 displayed the smallest effective joining area, and joint J6 had an intermediate value, which was in line with the mechanical performance exhibited by each joint. On the one hand, the observed gaps might be explained by the major differences in the thermo-mechanical behavior of the base materials (enumerated in Table 2), leading to different heating and cooling rates (identified in Figure 4), that may induce geometrical incompatibilities along the interface throughout the joining process, as identified by Li et al. [50]. On the other hand, since the experimental procedure was designed so that the process was position-controlled, another possible explanation for these gaps might be related to the trade-off between the vertical position, which was kept constant, and the vertical force, which might have decreased to keep the vertical position.

**Figure 7:**
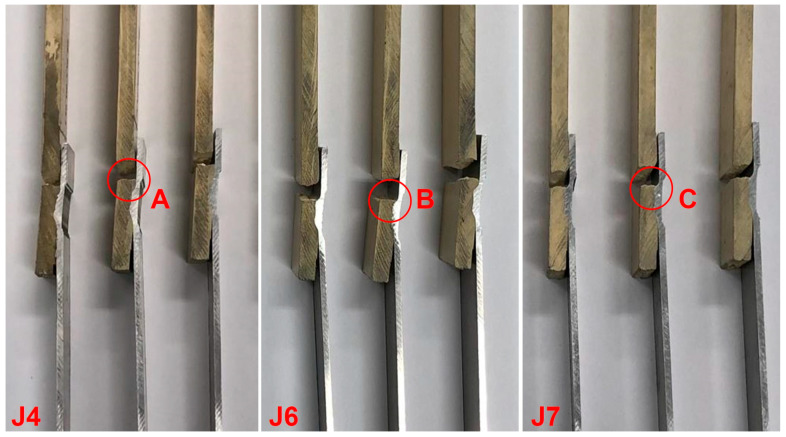
Fractured specimens associated with joints J4, J6, and J7.

Additionally, the previously mentioned microstructural gaps may also provide an explanation for the fracture site of the specimens. The failure of the specimens occurred on the AS of the overlap area in the transition between the composite joint region and the polymeric base material, as observable in details A, B, and C in Figure 7. This behavior was already reported by Correia et al. [40], who indicated that the resulting composite joints’ geometries significantly affected the amount of secondary bending moment generated due to the base materials’ neutral line misalignment. Consequently, as the levels of secondary bending moment increase, the bending stress also increases, reaching the UTS of Noryl^TM^ at lower values of UTL. Moreover, the fracture region in joint J7 was found to be located between positions D and E (refer to Figure 6 and Figure 7 for further details), where the cross-sectional area of the polymeric plate is decreased because of the aluminum plate clinching, further decreasing the UTL recorded in these joints. In this way, the differences in the mechanical strengths of the fabricated composite joints can be directly attributed to the effective joining area, but also indirectly to the development of secondary bending moments within the joining regions and the decrease in the cross-sectional area of the polymeric plate next to these regions.

## 4. Conclusions

In the present research study, a full factorial design of experiments was carried out to thermo-mechanically characterize AA6082-T6 to Noryl^TM^ GFN2 friction stir composite joints in overlap configuration, with the following highlights:

- After statistical analysis, it was possible to mathematically describe and predict both the mechanical strength (UTL) and processing temperature (using Tas as proxy) as a function of the processing parameters—rotational speed (ω), travel speed (v), and the tilt angle (α). The models were validated after comparing the predicted values, given by the numerical expressions, with the experimental ones. The resulting errors were found to be 0.7% of the UTL prediction and 2.3% of the average Tas prediction.
- From the thermal history of all fabricated joints, it could be concluded that processing temperatures between the softening and melting temperatures of Noryl^TM^ enable the development of proper binding mechanisms, resulting in significant joint strength.
- Joint strength was found to be mainly governed by the effective joining area, which might be considerably reduced by micro-gaps between base materials along the composite interface.
- The hardness profiles were found to be similar among the analyzed joints. The measurements taken over the aluminum lines revealed a noticeable decrease in hardness toward the SZ, with the minimum values observed in the TMAZ, forming the characteristic W-shaped profile. The hardness measurements over Noryl^TM^ did not evidence a significant change regardless of region and joint under analysis.

## Figures and Tables

**Table 1:** Joint efficiency of dissimilar joints using friction stir-based technologies.

Base Materials	AA6082 Noryl^TM^	AA6061 PEEK	AA6061 PC	AA5058 PMMA	AA5052 PP	AA5058 PC
Joint efficiency	47%	20%	35%	60%	17%	70%
Reference	[19]	[28]	[29]	[33]	[37]	[38]

**Table 2:** Main thermo-mechanical and physical properties of the base materials.

Material	E (GPa)	ρ (g/cm^3^)	UTS (MPa)	ε (%)	K (W/(m°C))	T_melt_ (°C)
AA6082-T6	70	2.70	* 290	10	180	580
Noryl	7	1.25	80	2.5	0.26	** 280

* Yield strength: 250 MPa ** T_soft_: 143 °C.

**Table 3:** FFD factors, symbols, and levels.

Factor	Symbol	Unit	Level 1	Level 2
Rotational speed	ω	rpm	600	1000
Travel speed	v	mm/min	100	140
Tilt angle	α	◦	2	3

**Table 4:** Summary of the FFD tensile shear test results and recorded processing temperature.

	Joint ID	ω (rpm)	v (mm/min)	α (◦)	Avg. UTL (N)
FFD joints	J1	600	100	2	2294.8 ± 215.2
	J2	1000	100	2	1749.9 ± 174.7
	J3	600	140	2	2168.8 ± 222.4
	J4	1000	140	2	3414.2 ± 317.1
	J5	600	100	3	1789.6 ± 58.7
	J6	1000	100	3	2323.6 ± 244.7
	J7	600	140	3	1708.1 ± 45.5
	J8	1000	140	3	1845.2 ± 42.4
Validation joint	J9	800	120	2.5	2350.0 ± 192.4

**Table 5:** Quasi-static tensile shear testing variance analysis—ANOVA.

Source	DF	SS	MS	F-Value	*p*-Value	Contribution
ω	1	666,511	666,511	14.60	0.001	7.0%
v	1	180,533	180,533	3.95	0.061	1.9%
α	1	1,892,478	1,892,478	41.45	<0.001	20.0%
ω·v	1	996,732	996,732	21.83	<0.001	10.5%
ω·α	1	23,862	23,862	0.52	0.479	0.3%
v·α	1	2,399,492	2,399,492	52.56	<0.001	25.4%
ω· v·α	1	2,427,639	2,427,639	53.17	<0.001	25.7%
Error	19	867,433	45,654			9.2%
Total	26	9,454,679				
R^2^ = 90.8%, adjusted R^2^ = 87.5%, predicted R^2^ = 80.2%						

**Table 6:** Average processing temperature on AS and RS of each composite joint.

Joint ID	ω (rpm)	v (mm/min)	α (◦)	Avg. Tas * (°C)	Avg. Trs ** (°C)
J1	600	100	2	243.6 ± 7.2	240.2 ± 8.4
J2	1000	100	2	253.9 ± 11.5	236.8 ± 17.2
J3	600	140	2	241.5 ± 14.6	183.7 ± 15.8
J4	1000	140	2	251.5 ± 9.7	247.0 ± 27.3
J5	600	100	3	203.6 ± 10.7	164.5 ± 20.1
J6	1000	100	3	228.6 ± 11.8	172.8 ± 25.3
J7	600	140	3	224.1 ± 12.2	193.7 ± 19.2
J8	1000	140	3	241.1 ± 14.7	186.8 ± 11.1
J9	800	120	2.5	232.2 ± 20.9	212.1 ± 15.1

* Tas: temperature on AS, ** Trs: temperature on RS.

**Table 7:** Advancing side temperature analysis of variance—ANOVA.

Source	DF	SS	MS	F-Value	*p*-Value	Contribribution
ω	1	485.76	485.76	15.52	0.017	24.7%
v	1	101.22	101.22	3.23	0.146	5.1%
α	1	1083.63	1083.63	34.63	0.004	55.0%
v·α	1	174.81	174.81	5.59	0.077	8.9%
Error	4	125.16	31.29			6.4%
Total	8	1970.58				
R^2^ = 93.7%, adjusted R^2^ = 87.3%, predicted R^2^ = 70.0%						

## Data Availability

Data are available upon request to the corresponding author.
